# 3D-Printable Sustainable Bioplastics from Gluten and Keratin

**DOI:** 10.3390/gels10020136

**Published:** 2024-02-07

**Authors:** Jumana Rashid Mohammed Haroub Alshehhi, Nisal Wanasingha, Rajkamal Balu, Jitendra Mata, Kalpit Shah, Naba K. Dutta, Namita Roy Choudhury

**Affiliations:** 1 Chemical and Environmental Engineering, School of Engineering, STEM College, RMIT University, Melbourne, VIC 3000, Australia; s3577645@student.rmit.edu.au (J.R.M.H.A.); rajkamal.balu@rmit.edu.au (R.B.); kalpit.shah@rmit.edu.au (K.S.); 2 Australian Centre for Neutron Scattering (ACNS), Australian Nuclear Science and Technology Organisation (ANSTO), Lucas Heights, NSW 2232, Australia; jitendra.mata@ansto.gov.au; 3 School of Chemistry, University of New South Wales, Sydney, NSW 2052, Australia

**Keywords:** film, protein, gluten, keratin, neutron scattering, 3D printing

## Abstract

Bioplastic films comprising both plant- and animal-derived proteins have the potential to integrate the optimal characteristics inherent to the specific domain, which offers enormous potential to develop polymer alternatives to petroleum-based plastic. Herein, we present a facile strategy to develop hybrid films comprised of both wheat gluten and wool keratin proteins for the first time, employing a ruthenium-based photocrosslinking strategy. This approach addresses the demand for sustainable materials, reducing the environmental impact by using proteins from renewable and biodegradable sources. Gluten film was fabricated from an alcohol–water mixture soluble fraction, largely comprised of gliadin proteins. Co-crosslinking hydrolyzed low-molecular-weight keratin with gluten enhanced its hydrophilic properties and enabled the tuning of its physicochemical properties. Furthermore, the hierarchical structure of the fabricated films was studied using neutron scattering techniques, which revealed the presence of both hydrophobic and hydrophilic nanodomains, gliadin nanoclusters, and interconnected micropores in the matrix. The films exhibited a largely (>40%) β-sheet secondary structure, with diminishing gliadin aggregate intensity and increasing micropore size (from 1.2 to 2.2 µm) with an increase in keratin content. The hybrid films displayed improved molecular chain mobility, as evidenced by the decrease in the glass-transition temperature from ~179.7 °C to ~173.5 °C. Amongst the fabricated films, the G14K6 hybrid sample showed superior water uptake (6.80% after 30 days) compared to the pristine G20 sample (1.04%). The suitability of the developed system for multilayer 3D printing has also been demonstrated, with the 10-layer 3D-printed film exhibiting >92% accuracy, which has the potential for use in packaging, agricultural, and biomedical applications.

## 1. Introduction

The pursuit of sustainable materials to address the environmental challenges posed by non-degradable plastics has led to the exploration of bioplastics as a viable alternative. Bioplastics, which is made from renewable materials, are seen as a component of a future circular economy that contributes to the attainment of the United Nations’ (UN) Sustainable Development Goals. Amongst various routes, one promising avenue in this endeavor involves the development of hybrid bioplastic films that combine both plant-derived and animal-derived proteins, thereby harnessing the strengths of both domains. For example, plant-derived proteins, such as wheat gluten, are renowned for their biodegradability and cost-efficiency [1,2]. Hybrid bioplastics fabricated using these proteins exhibit enhanced physical properties, making them a versatile candidate for diverse applications. In the packaging industry, such bioplastics can potentially offer superior barrier properties as compared to conventional plastics, a critical attribute for prolonging shelf life and maintaining product quality [3,4]. Among the biodegradable protein polymers, plant gluten and animal keratin have emerged as prominent candidates, offering immense potential to provide eco-friendly alternatives to petroleum-based plastics, which can be tailored to undergo tunable biodegradation [5]. Material biodegradation rates are influenced by various factors, including moisture and oxygen permeability, hydrophobicity, and the chemical composition of employed polymers, in addition to the actual method of biodegradation [6]. The capacity to tailor the biodegradability and physicochemical characteristics of biopolymer-based materials holds substantial promise, especially in fields like packaging, biomedicine, and agriculture. By amalgamating hydrophobic and hydrophilic components, we can regulate the degradation kinetics within the resulting composites [7]. This rationale underscores the significance of utilizing gluten, a naturally occurring protein present in grains including wheat, barley, and rye, which is being explored for its potential use in bioplastics. However, the intrinsic hydrophobic nature of gluten poses a pronounced challenge concerning environmental degradation [8].

**Figure 1:**
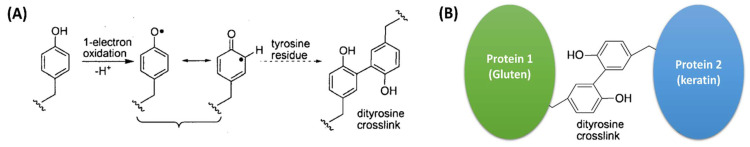
(**A**) Mechanism of tyrosine cross-linking, and (**B**) intermolecular dityrosine cross-linking in proteins.

Gluten is comprised of two primary constituents: (i) gliadin, which readily dissolves in alcohol–water mixtures, and (ii) glutenin, characterized by limited solubility due to its intricate cross-linked structure [9]. To enhance its biodegradability for applications in bioplastics and agricultural films, it is imperative to fractionate soluble gluten and combine it with hydrophilic materials. On the other hand, keratin is a type of fibrous protein that forms filaments. It is categorized into α and β types and is commonly found in hair, nails, and feathers [10] and is capable of the formation of disulfide bonds, primarily facilitated by sulfur-containing amino acids like cysteine [2]. Due to its excellent film-forming characteristics in the presence of glycerol, prior research has explored keratin in the context of biopolymer films by blending hydrolyzed keratin with a diverse array of materials [2,11,12]. The inherent biodegradability and abundance of gluten and keratin make them promising candidates for sustainable materials. However, gluten/keratin hybrid blends have not been investigated in the literature, and the challenge lies in effectively combining these proteins to create bioplastics with enhanced properties. In this study, we bridge this research gap by using the unique approach of crosslinking gluten and keratin using a tyrosine-based strategy, where both proteins contain tyrosine residues [13,14]. The overarching goal is to investigate the synergistic fusion of keratin with gluten proteins through a distinctive tyrosine photocrosslinking method. This innovative approach holds the potential to unlock new avenues for sustainable applications of gluten- and keratin-based bioplastic films. The photocrosslinking strategy (Figure 1) also adds an extra layer of versatility, enabling the application of the resulting bioplastic films as a sprayable or printable coating of any desired shape. Thus, our aim is to examine the synergistic fusion of keratin with gluten proteins using a unique tyrosine photocrosslinking approach, resulting in protein-based bioplastic films with the potential to fully exploit these proteins’ capabilities for sustainable applications. The rapid crosslinking methodology employed using a ruthenium-based photocrosslinking strategy (Figure 1) also enables it to be applied as a spray or for printing to derive the resultant coating or film of any desired shape, where the rheological properties of the protein inks are crucial for successful spraying or 3D printing. However, integrating this technology with bioplastic materials, particularly those derived from proteins like gluten and keratin, introduces specific challenges and limitations.

Only a very few studies have reported 3D printing of gluten- and keratin-based materials in the literature. For example, oxidized human hair keratin constructs have been 3D printed using a photosensitive ink; however, they required a total crosslinking time of 20 min [15]. Conversely, gluten has only been 3D printed in combination with other proteins like soy, also in the presence of thermosensitive additives [16]. One of the primary challenges in 3D printing bioplastic films is achieving the necessary balance between material flow properties and structural integrity. The rheological behavior of bioplastic inks, crucial for successful extrusion and shaping, often requires precise tuning to facilitate proper layering and adhesion during printing [16]. Inconsistent material viscosity can lead to poor resolution, structural weaknesses, and inaccuracies in the final print [17]. However, the correlation between the rheological properties and the capacity to be 3D printed has not been investigated for these fusion protein systems. In this work, a series of hybrid systems composed of gluten and keratin in different weight ratios have been prepared. The developed systems have been extensively characterized using spectroscopy, microscopy, scattering, and rheological investigations, and finally, their 3D printability has been examined to ensure they are suitable for food packaging, biosensors, and bioplastic films [18]. Another significant challenge is the inherent limitations of bioplastic materials in terms of mechanical strength and durability, especially when compared to conventional plastic materials [17]. The environmental conditions during the printing process, such as temperature and humidity, can significantly affect the properties of bioplastics, potentially leading to deformations or compromised material quality [19].

To address these challenges, several solutions have been proposed and are under active research. Material modification techniques, such as the incorporation of crosslinking agents or blending with other polymers, have been explored to enhance the mechanical properties of bioplastic films [20,21]. This approach seeks to improve the tensile strength and flexibility of the printed objects, making them more suitable for a wider range of applications. The development of advanced printing techniques and equipment is another avenue for overcoming these challenges. For instance, implementing precise temperature control during the printing process can significantly improve the consistency and quality of bioplastic films [22]. Additionally, the use of specialized nozzles and optimized printing speeds can enhance the resolution and accuracy of the printed objects [23]. Moreover, post-processing treatments such as UV curing, annealing, or chemical treatment can be employed to further strengthen printed bioplastic films, enhancing their durability and functional performance [24]. These treatments can help to solidify the structure, close micropores, and improve the overall resilience of the material. However, the primary challenge lies in achieving precise 3D printing of chemically cross-linked gluten and keratin composite gels or films while maintaining both a high resolution and structural integrity [25]. With the aim of obtaining in-depth insights into the printability of these fused protein–protein films, the present investigation focuses on the development of a family of 3D-printable gel films composed of gluten and keratin proteins from biomass sources. Through this research, we seek to investigate the potential of gluten and keratin as critical components in tuning physicochemical properties and degradability.

## 2. Results and Discussion

### 2.1. Secondary Structure and Molecular Chain Mobility of Gluten/Keratin Hybrid Film

**Figure 2:**
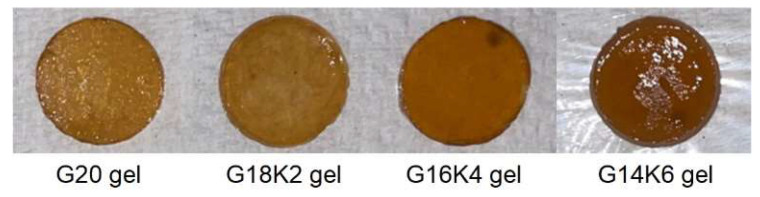
Photographs of photocrosslinked gel films.

The molecular chain mobility and water degradation profile of protein-based films are largely influenced by the secondary structure of proteins, which can be studied using FTIR spectroscopy, allowing for both qualitative and quantitative analyses. The keratin used in this study is a hydrolyzed protein with a relatively low molecular weight of 1464 Da (derived by matrix-assisted laser desorption/ionization-time of flight mass spectrometry; refer to the Supplementary Materials for further information.). Moreover, hydrolyzed low-molecular-weight keratin has been previously reported to adopt a random coil secondary structure conformation in aqueous solutions [26]. On the other hand, commercially available wheat gluten protein has been reported to largely exhibit α-helix β-sheet secondary structures [27]. Figure 2 shows the photographs of fabricated gel films, and their material composition is given in Table 1.

**Figure 3:**
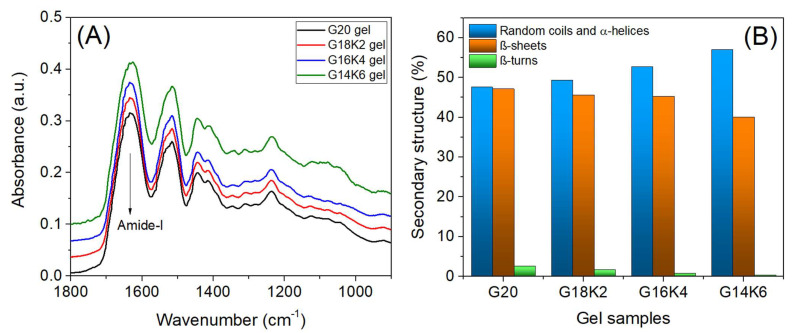
(**A**) FTIR analysis of gluten/keratin hybrid films and (**B**) quantitative analysis of secondary structure.

Figure 3A shows the FTIR spectra of fabricated films. All fabricated films exhibited clear spectral patterns, including two notable peaks in the frequency range of 1500−1600 and 1600−1700 cm^−1^ corresponding to amide-II and amide-I protein conformational bands, respectively. In order to quantitatively estimate the different secondary structures of proteins in the films, the background subtracted amide-I band (1580–1690 cm^−1^) of the FTIR spectra was deconvoluted (see Supplementary Materials for more details) using wavenumbers corresponding to different conformational elements, such as β-sheets (1684 cm^−1^), β-turns (1671 cm^−1^), random coil and α-helices (1650 cm^−1^), intramolecular β-sheets (1631 cm^−1^), intermolecular β-sheets (1615 cm^−1^), and the glutamine side chain (1596 cm^−1^) [28]. The estimated secondary structure percentages (obtained from the deconvoluted peak area) are presented in Figure 3B. The pristine gluten film (G20) exhibited 47.15% β-sheets, 47.58% random coils and α- helix, and 2.67% β-turns, which is generally consistent with reports in the literature [27]. The films made from a combination of gluten and keratin showed a rise in random coil and α-helix structures as the amount of keratin increased. This observation is ascribed to the fact that the hydrolyzed keratin protein tends to form random coil structures. The FTIR results further support the trend observed in water uptake and rheology measurements.

**Figure 4:**
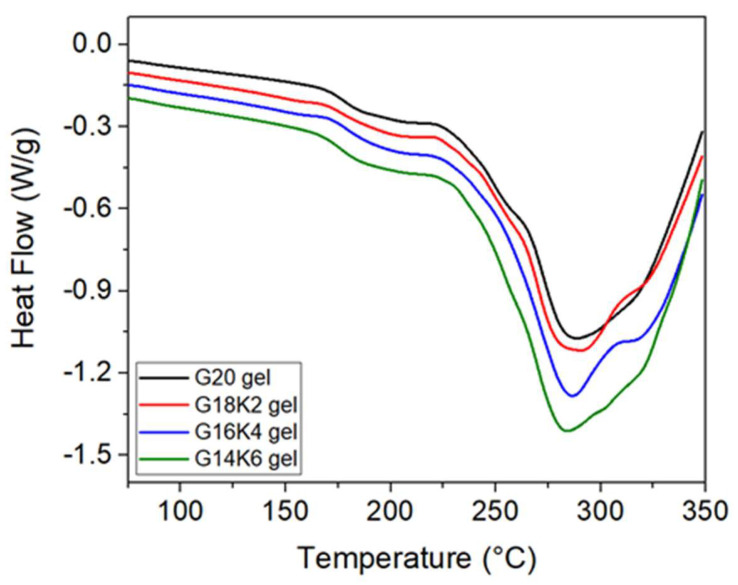
DSC thermograms of fabricated gels.

The impact of keratin content on the molecular chain mobility of gluten/keratin hybrid films was analyzed using the DSC technique. The DSC thermograms of freeze-dried gels (Figure 4) show two significant thermal transitions: (i) a shift in baseline related to the glass-transition temperature (T_g_) and (ii) a complex endothermic peak above 275 °C related to thermochemical degradation. The pristine gluten gel (G20) exhibits a T_g_ value of ~179.7 °C, which is in the range reported in the literature for wheat glutenin and gliadin proteins [29]. An increase in keratin content resulted in a systematic decrease in T_g_ of the hybrid gels (178.3 °C for G18K2, ~177.2 °C for G16K4, and ~173.5 °C for G14K6), which may be due to the low T_g_ value of ~108.1 °C for hydrolyzed keratin. The observed trend in the molecular chain mobility of films is in good agreement with that observed for the secondary structure. The decrease in the T_g_ value suggests an increase in molecular chain mobility, where the G14K6 hybrid gel exhibited the highest molecular chain mobility.

### 2.2. Water Uptake Behavior and Morphology of Gluten/Keratin Hybrid Film

The water absorption property and microstructure of the film largely influence their physicochemical properties and degradability. By characterizing the equilibrium water uptake capacity and micropore size/shape of the fabricated films, we can gain insight into the effect of varying proportions of gluten and keratin proteins on the crosslink density, viscoelastic properties, and hydrolytic degradation of the hybrid gels [30]. Understanding the relationship between these properties can assist in creating innovative gels and films with customized characteristics. The crosslink density and water absorption capacity of the films, computed via Equations (2)–(4), are presented in Table 1. The pristine gluten film shows a water uptake capacity of ~83% and a crosslink density of ~0.365 mol/cm^3^. A systematic increase in water uptake was observed with a progressive increase in keratin content, with 181% (more than double that of gluten) water taken up at a 70:30 gluten: keratin ratio. The findings demonstrate that films with higher concentrations of keratin proteins exhibited enhanced water absorption capabilities. This observation can be attributed to the presence of hydrophilic groups in the matrix with an increase in keratin content. The observed increase in the water uptake value of the films upon the inclusion of keratin may also be influenced by the interplay between the hydrophilic head (-SO^4−^) of keratin and the ionic groups present in gluten macromolecules, specifically the quaternary ammonium groups derived from histidines, lysines, and arginines [2,10,31]. Notably, this study reveals a correlation between the crosslink density and the water absorption capacity of the films. Despite the lack of significant changes in crosslink density, the observed correlation suggests that additional factors contribute to the variations in the water absorption capacity of the fabricated films.

**Figure 5:**
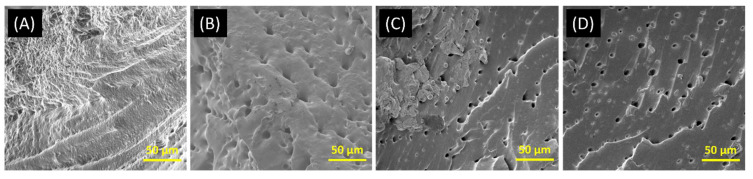
SEM images of (**A**) G20, (**B**) G18K2, (**C**) G16K4, and (**D**) G14K6 gels.

The water absorption behavior of the hybrid film is seen to exhibit a non-Fickian behavior, indicating that water transfer within the films is not solely governed by diffusion. The observed behavior can be ascribed to the existence of the crosslinked network structure of the gel films, which facilitates the movement of water through convection [22]. Keratin, as a proteinaceous constituent, can establish intermolecular cross-linkages with gluten proteins, thereby modifying the structure of the gel network and facilitating the development of a more permeable architecture. The inclusion of keratin in the gluten matrix results in the disruption of its compactness, thereby facilitating the formation of interconnected pores and void spaces [32]. SEM results (Figure 5) also show evidence of increased pore size (Table 1) with an increase in keratin content.

### 2.3. Hierarchical Structure of Equilibrium Water Swollen Gluten/Keratin Hybrid Film

**Figure 6:**
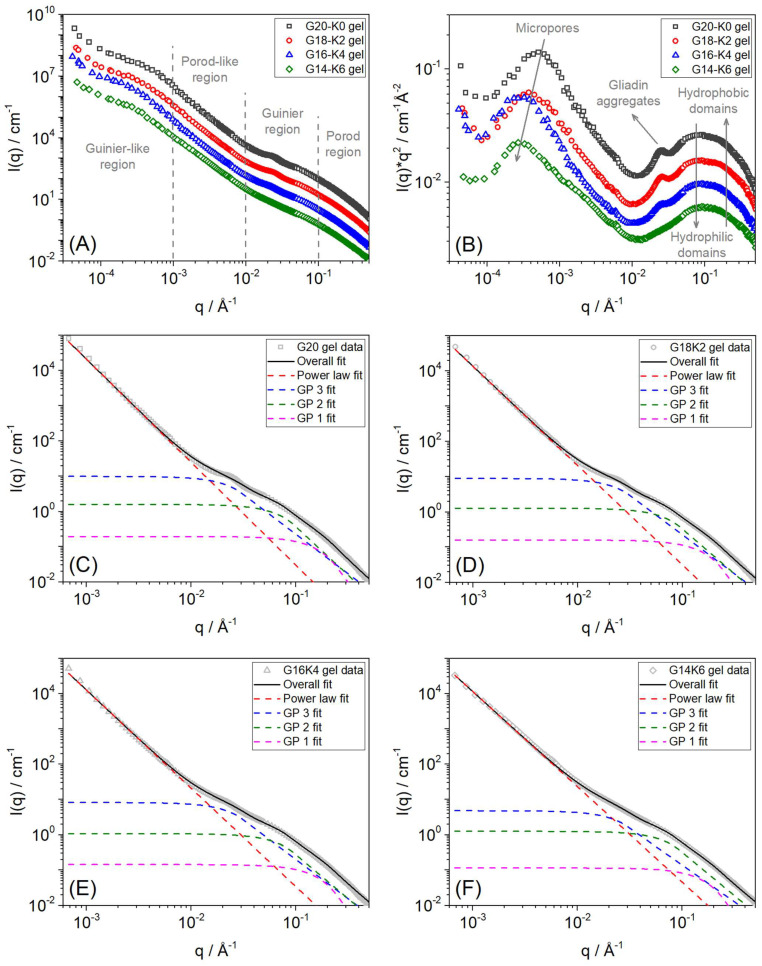
(**A**) SANS intensity profile and (**B**) Kratky plot of the four fabricated gluten/keratin hybrid films/gels (G20, G18K2, G16K4, and G14K6), presented with intensity offset for clarity. Shape-independent form factor models (power law + three Guinier−Porod (GP)) fit to SANS data of (**C**) G20, (**D**) G18K2, (**E**) G16K4, and (**F**) G14K6 films/gels.

The physicochemical properties of films are transmitted across various-length scales in the polymer matrix. Therefore, understanding the hierarchical structure of films is crucial to tuning their water swelling, viscoelastic, mechanical, and degradation properties. SANS and USANS are powerful techniques to study the hierarchical structure of protein-based systems from dilute solutions to crosslinked films, even in a highly crowded condition across length scales from a few angstroms to several micrometers within the protein/polymer network [33,34,35]. In this study, for the first time, a neutron scattering experiment on gluten/keratin-based protein gels has been conducted covering five decades of length scale. Resulting SANS and USANS data were used to analyze the hierarchical organization and internal structure of the fabricated films in the size range of 9.0 Å to 12.6 µm in unprecedented detail. Figure 6A illustrates and compares the SANS and USANS intensity profiles of the fabricated films equilibrium swollen in D_2_O. The plots show three distinctive q-regions in the SANS data (a high-q Porod region from 0.1 to 0.5 Å^−1^, a mid-q Guinier region from 0.01 to 0.1 Å^−1^, and a low-q Porod-like region from 0.001 to 0.01 Å^−1^), and one in the USANS data (a very low-q Guinier-like region). These regions can be clearly identified in the Kratky plot (Figure 6B), which divides the decay of the scattering at high-q and shows the scattering features more prominently [36]. The four clearly visible peak features from high-q to very low-q can be attributed to (i) the hydrophobic domain, (ii) the hydrophilic domain, (iii) gliadin (water-soluble protein fraction of wheat gluten) aggregates, and (iv) micropores in the films [37,38]. Furthermore, for protein-based film systems, the slope value estimated from the high-q Porod region and low-q Porod-like region can provide information about the “fractal dimension” of the intrinsic structure and the quality of the interface of the protein molecules and their cross-linked microporous network structures, respectively [33]. On the other hand, the mid-q Guinier region and very low-q Guinier-like region can provide information about the size, shape, and correlation length of the internal domains and micropore size, respectively. Therefore, to estimate the structural parameters of the fabricated films, a combined shape-independent form factor model comprising a power law and three Guinier–Porod models was fit to the experimental SANS data, as shown in Figure 6C–F. The Guinier–Porod model (given below) estimates the size and dimensionality of generalized Guinier/power law scattering objects [39].

(1) $$I\left(q\right)=Gexp\left(\frac{-q^{2}{R_{g}}^{2}}{3}\right)\;for\;q\leq q_{1};\;I\left(q\right)=\left(\frac{D}{q^{d}}\right)\;for\;q\geq q_{1}$$

where *q* is the scattering variable, *I*(*q*) is the scattered intensity, *R_g_* is the radius of gyration, d is the Porod exponent, *G* is the Guinier scale factor, and *D* is the Porod scale factor. The structural parameters determined from the model fits are given in Table 2.

As wheat gluten is made up of globular proteins, the dimension variable (s) was fixed as zero for Guinier−Porod (GP) model fitting [39]. The high-q GP 1 fit returned a Porod slope and R_g_ of ~4.0 and ~10.0 Å, respectively, for all the fabricated films, which can be attributed to the β-sheet secondary structure (i.e., hydrophobic domain exhibiting sharp interface with surrounding medium/matrix) of the globular protein. The mid-q GP 2 fit returned Porod slope and Rg values of around 2.5 and 20.5 Å for all the fabricated films, which can be attributed to hydrophilic domain (or correlation length between β-sheet structures) with mass fractal-like dimensions. On the other hand, the mid-q GP 3 fit returned Porod slope and R_g_ values of around 2.25 and 59.0 Å for all the fabricated films, which can be attributed to gliadin aggregates in the matrix with mass fractal-like structures. With an increase in the keratin concentration, the intensity of the gliadin aggregates’ scattering was observed to systematically decrease in films, indicating better solubility or the disruption of aggregates with keratin in hybrid films via inter-molecular interactions. The low-q power law fit of G20 film returned a Porod slope of around 2.9, which decreased to 2.6 with 30 wt.% keratin substitution, suggesting loose network formation and the anticipated decrease in mechanical properties. This is also supported by the increased trend in estimated micropore size (calculated from the very low-q USANS data using the relation d = 2π/q, where d is the peak value in the Kratky plot) and the measured water uptake. The micropore sizes (1.2 to 2.2 µm) estimated from USANS data also generally agree with values obtained from SEM results. Moreover, the absence of an intensity plateau in the very low-q region indicates that the size and structure of the fabricated films are beyond the USANS measurement range.

### 2.4. Viscoelastic Properties of Gluten/Keratin Hybrid Film

**Figure 7:**
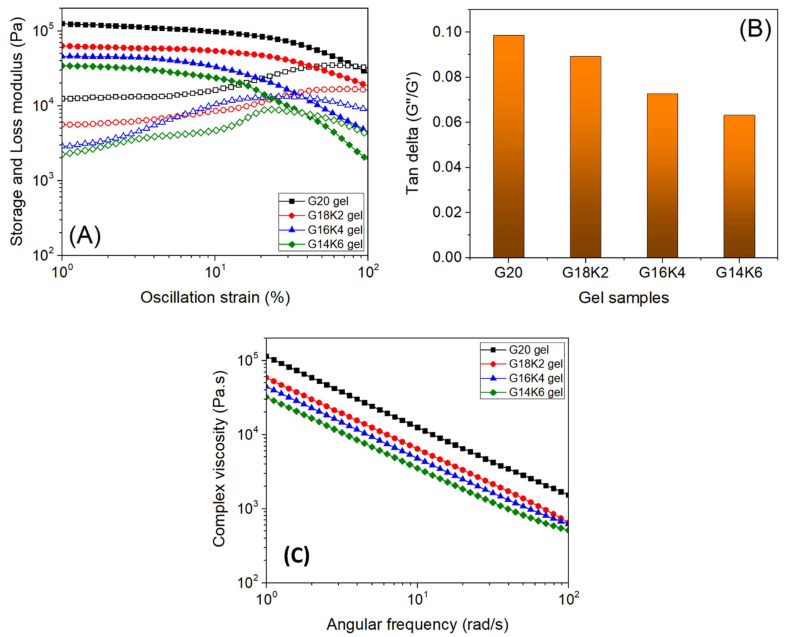
(**A**) Storage (G′, filled symbols) and loss modulus (G″, open symbols) of films measured as a function of oscillation strain, (**B**) tan delta of films measured at linear viscoelastic regime, and (**C**) complex viscosity of films measured as a function of angular frequency.

Protein-based films exhibit complex viscoelastic behavior, which is largely influenced by their microstructure, crosslink density, and level of hydration [40]. Figure 7A shows the G′ (elastic) and G″ (viscous) responses of fabricated gel films measured as a function of oscillation strain. All films exhibit a linear viscoelastic regime (LVR, plateau region) at low strain, followed by a network rupture/deformation regime (slope region) at high strain. The LVR is important in dynamic rheological analysis because the viscoelastic parameters are strain-independent at LVR [41]. The G′ value of all films at LVR is approximately one order of magnitude higher than G″, suggesting that the film’s viscoelastic properties are primarily governed by an elastic response.

The G20 film exhibited the largest LVR and highest G′ among the fabricated films, whereas the LVR and G′ systematically decreased with an increase in keratin content for hybrid films. This observation indicates that the hybrid films become softer with an increase in keratin content. In addition, the tan delta (G″/G′) values of hybrid films at LVR (Figure 7B) show a decreasing trend with keratin content, suggesting the films become less energy dissipative or more elastic, which can be attributed to the trend observed for crosslink density and water uptake (where water acts as a plasticizer) [42,43]. Moreover, the complex viscosity of all the films exhibits shearthinning behavior (a decrease in viscosity with an increase in frequency, as shown in Figure 7C), which suggests the suitability of the developed films for printing applications [44] and the extrudability of the material from the nozzle easily during 3D printing. The materials’ shear thinning nature facilitates a more efficient extrusion process during 3D printing. As the shear rate escalates, the viscosity of the material decreases, ensuring a smooth and consistent flow through the printer nozzle [45]. This controlled reduction in viscosity is fundamental for precise layer-by-layer deposition, contributing to the accuracy and fidelity of the final printed object. Moreover, shear thinning enhances the printability of the materials by making them more responsive to the shear forces inherent in the 3D printing process [46]. This responsiveness is pivotal for achieving controlled and precise shaping of the material during extrusion, resulting in improved print quality and structural integrity. This fine-tuned control is critical for achieving uniform layer thickness and adherence, influencing the overall stability and quality of the printed object. The rapid recovery of viscosity after shear stress ensures the immediate stabilization of extruded layers [47]. This swift transition from lower to higher viscosity contributes to layer adhesion, maintaining the shape and structure of each layer during the printing process. The differences in complex viscosity may be related to the composition and structural change in the films with crosslink density.

### 2.5. Hydrolytic Degradation of Gluten/Keratin Hybrid Film

**Figure 8:**
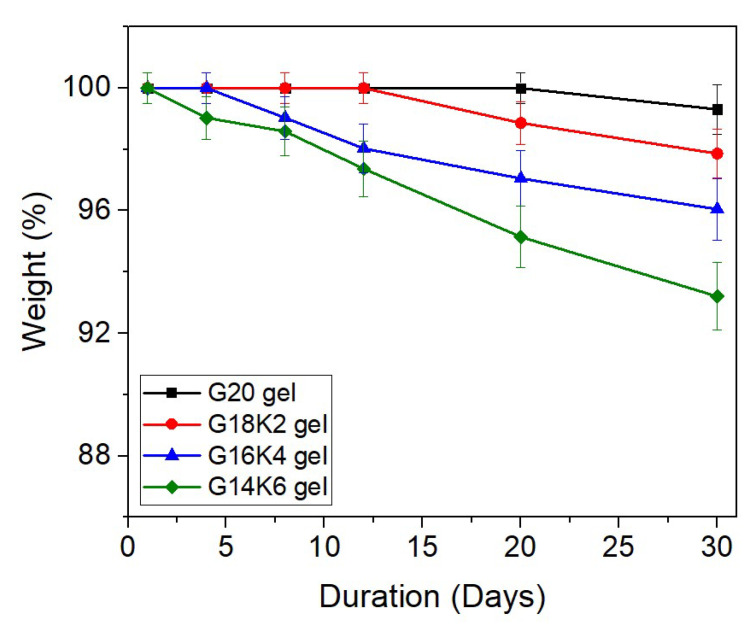
Hydrolytic degradation behavior of fabricated film.

Protein-based films undergo hydrolytic degradation, where peptide bonds undergo hydrolysis when they encounter water molecules, resulting in their fragmentation and the generation of new terminations in the chain [29]. Therefore, the study of the water degradation behavior of fabricated crosslinked films is crucial for understanding their long-term performance and suitability for various applications [48]. Figure 8 shows the effect of keratin content on the in vitro hydrolytic degradation (at 25 °C) behavior of gluten/keratin hybrid films over a period of one month. The gluten film exhibits excellent water resistivity, with no significant weight loss observed for the entire study duration. This can be attributed to the large gliadin fraction present in the gluten film, a property highly valuable in various applications like food packaging. Gliadin’s hydrophobic amino acids, like proline, create a water-repelling structure, and its protein network acts as a barrier to water infiltration [49]. Additionally, gliadin lacks hydrophilic sites that readily interact with water molecules, enhancing the film’s hydrophobicity. Combined with specific processing techniques, these factors contribute to the exceptional water resistivity of gluten films, making them a sustainable choice for industries requiring protection against moisture [50]. The hybrid films demonstrated a consistent reduction in water resistivity as the keratin content increased, which can be attributed to the more hydrophilic nature of keratin [51] and the observed trend in crosslink density. The G14K6 film, which exhibited the highest water uptake capacity as shown in Table 1, showed noticeable weight loss from day 4 onwards, which reached around 6.8% weight loss after 30 days. The G20, G18K2, and G16K4 gels exhibited weight losses of around 0.7%, 2.2%, and 4.0%, respectively, for the same duration. The observed trend in the hydrolytic degradation of films is in good agreement with the trend observed for the secondary structure (Table 3). Therefore, it is evident that hydrolyzed keratin can be effectively used to tune the hydrolytic degradation profile of the gluten film.

### 2.6. 3D Printing of Gluten/Keratin Hybrids

**Figure 9:**
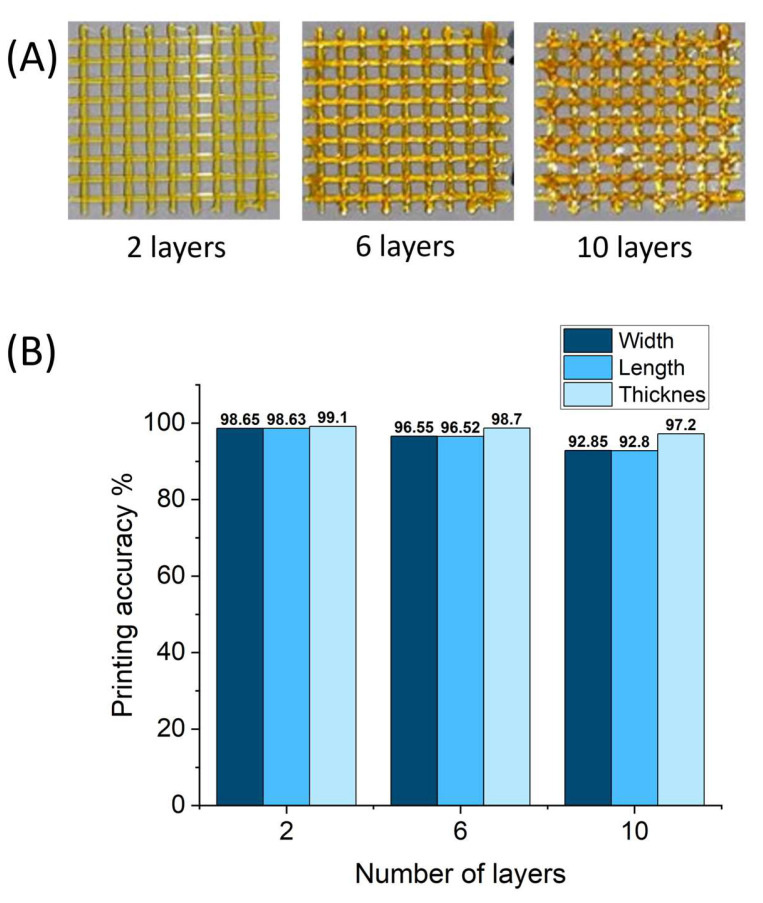
(**A**) Photographs of 3D-printed grid structures and (**B**) corresponding printing accuracy of gluten/keratin (G14K6) hybrid gels.

To evaluate the printability of the developed gluten/keratin hybrid film system, photocrosslinkable ink comprising gluten and keratin in a weight ratio of 70:30 (precursor for G14K6 gel, which exhibited the highest water uptake capacity among the fabricated film) was layer-by-layer 3D printed (up to 10 layers) into a grid structure, as shown in Figure 9A. The 3D printing was performed with optimized printing parameters (pneumatic pressure and print speed of 60 kPa and 17.5 mm/s, respectively), and each printed layer was photocured subsequently. Figure 9B shows the calculated printing accuracy for width, length, and thickness (using Equation (5) of 2-, 6-, and 10-layer grid structures. The 2-layer grid structure exhibited a printing accuracy of >98%, which decreased systematically with an increase in printing layers. This can be attributed to interaction-driven adjacent filament fusion and gravity-driven deformation of the filament along the axial direction with an increase in height [52]. Nevertheless, the 10-layer 3D printed grid structure of the hybrid film exhibited a printing accuracy of >92% for its width and length, which is significantly higher than previously reported 3D-printable photocrosslinked silk fibroin and soy protein-based film systems [53]. The higher printing accuracy provides an excellent opportunity for the fabrication of complex 3D structures, which have the potential to be utilized in packaging or biomedical fields.

In addition to the aforementioned results, it is crucial to discuss the interplay between the nozzle size and the filament dimensions in our 3D printing process. The experimental setup utilized a 23-gauge nozzle, corresponding to a diameter of approximately 0.643 mm. The filament, a photocrosslinkable ink comprising a gluten/keratin hybrid, was extruded with a thickness ranging between 0.58 and 0.61 mm. This close alignment between the filament thickness and the nozzle diameter is a key factor contributing to the high printing accuracy observed in the study. The slight oversizing of the nozzle compared to the filament thickness facilitates smooth extrusion while minimizing the risk of clogging, which is critical in maintaining consistent print quality, especially when printing complex structures [54,55,56]. The precision in filament extrusion, influenced by this nozzle-to-filament ratio, is particularly evident in the higher-layer structures. While the printing accuracy slightly decreased with an increase in the number of layers, possibly due to filament fusion and gravitational deformation, the overall high accuracy levels exceed those of other materials like silk fibroin and soy protein-based systems. This observation underscores the importance of optimizing the nozzle and filament dimensions to achieve superior printing outcomes, especially in applications demanding intricate detailing and high structural integrity, such as in packaging or biomedical fields. This scenario, where the extruded filament is slightly thinner than the nozzle diameter, is common in 3D printing and generally acceptable [54,57]. The deliberate choice of this configuration in our study aligns with industry practices and contributes to the smooth and reliable functioning of the 3D printing process.

**Table 3:** Results from this study compared with relevant literature reports.

Properties and Parameters		This Study (Gluten & Keratin)	Studies on Gluten [58,59,60]	Studies on Keratin [61,62,63]
Secondary structure from FTIR	β-sheets	40–47 (%)	47 (%)	28 (%)
	random coils and α- helix	47–57 (%)	45 (%)	65 (%)
	β-turns	0.4–2.7 (%)	8 (%)	7 (%)
Tg from DSC		173–180 (°C)	130–180 (°C)	150 (°C)
Water uptake capacity *		80–186 (%)	72 (%)	597 (%)
Micropore size from SEM		1–6 µm	-	-
Weight loss after 30 days from hydrolytic degradation		0.7–6.8 wt.%	-	-

* The outcomes could differ based on the length of water immersion and the study’s conditions, such as temperature, humidity, and pH levels.

## 3. Conclusions

In summary, we have successfully fabricated gluten/keratin hybrid films using a tyrosine-mediated photocrosslinking method. The hybrid films exhibit a tunable structure and properties based on the composition of the proteins. An increase in keratin content (up to 30 wt.%) in the hybrid films showed an increase in the hydrophilicity of the gels and resulted in increased random coil secondary structure, water uptake capacity, micropore size, and hydrolytic degradability, with decreased viscoelastic modulus and thermal stability. The hierarchical structure of these films, as revealed by neutron scattering, includes both hydrophobic and hydrophilic nanodomains, gliadin nanoclusters, and interconnected micropores, indicating a complex internal architecture. The hybrid films also demonstrate enhanced molecular chain mobility, evidenced by a decrease in the glass-transition temperature, which is particularly noticeable in the G14K6 variant. This variant also exhibits significantly higher water absorption (6.80% after 30 days) compared to the G20 variant (1.04%) Moreover, the hybrid systems exhibit good rheological properties suitable for 3D printing applications, achieving high accuracy (>92%) in printing up to 10 layers of the grid structure.

The development of gluten/keratin hybrid films for 3D printing presents significant opportunities for future exploration. Key areas of focus should include optimizing protein ratios for improved printing properties, conducting in-depth structural analyses, and evaluating long-term durability in diverse environments. Upscaling production and assessing mechanical properties tailored to 3D-printing applications are essential to extend their practical use. Additionally, investigating biofunctionalization, exploring alternative cross-linking agents, and a comprehensive environmental impact assessment through life cycle analysis are crucial. Emphasizing research tailored to specific applications and fostering interdisciplinary collaborations will be instrumental in maximizing the potential of these materials. Collectively, these efforts will advance the utility and sustainability of gluten/keratin hybrid films, particularly in their application to 3D printing accuracy and performance.

## 4. Materials and Methods

### 4.1. Materials

Gluten from wheat, ammonium persulfate (APS), and tris(2,2-bipyridyl)dichlororuthenium(II) hexahydrate (Ru(BPY)_3_) were procured from Sigma-Aldrich, Sydney, Australia. Keratin powder was purchased from Spectrum Chemicals, Australia. Isopropyl alcohol and ethanol were obtained from Chem-Supply, Adelaide, Australia.

### 4.2. Preparation of Fractionated Gluten Protein Solution

Glutenins and gliadins are two distinct families of proteins that collectively constitute a significant portion of the gluten component. While gliadins are relatively low molecular weight in the range of 28 to 55 kDa [64] and are soluble in alcohol–water mixtures [9], glutenins are of high molecular weight ranging from 30 to 140 kDa [65] and insoluble [66]. In this work, an alcohol–water mixture soluble gliadin protein fraction was first separated and purified. Briefly, 40 g of wheat gluten powder was added to 400 mL of a solvent mixture consisting of isopropanol and water at a 1:1 volume ratio and stirred for 12 h to obtain a uniform dispersion. The slurry was then centrifuged at 15,000× *g* for 30 min, and the obtained supernatant was refrigerated for 24 h. This led to the coacervation of gliadin molecules (around 30 wt.%) forming a high-density phase at the bottom, which was then separated and used for film fabrication.

### 4.3. Preparation of Keratin Protein Solution

A 60 wt.% keratin solution was prepared by dissolving 0.6 g of keratin powder in 1 mL of solvent consisting of isopropanol and water at a 1:1 volume ratio. The mixture was subjected to continuous stirring at 60 rpm and 20 °C for 24 h.

### 4.4. Fabrication of Gluten/Keratin Hybrid Films

The as-prepared gluten and keratin solutions were mixed (using a magnetic stirrer) at different weight ratios (Gluten:keratin of 100:0, 90:10, 80:20, and 70:30) for 3 h. To fabricate the film, a photocrosslinking method was employed using Ru(BPY)_3_ as the photocatalyst. An aqueous solution of Ru(BPY)_3_ (electron donor) was first added to the mixed protein solution and stirred for 10 min in the dark, followed by the addition of an aqueous APS (electron acceptor) solution and stirring in the dark for 5 min. The resultant solution combination was cautiously transferred into a Teflon mold, which was then exposed to a white light source with an intensity of 50 W for a duration of 2 min. The obtained cross-linked gel films (Figure 2) were dialyzed against Milli-Q water for 24 h to leach out the excess reactants and obtain equilibrium in the water uptake. To analyze the swelling ratio and other physicochemical properties, the films were subjected to oven-drying and freeze-drying, respectively.

### 4.5. Fourier-Transform Infrared (FTIR) Spectroscopy

FTIR analysis of the fabricated film was conducted in the dehydrated state to analyze the secondary conformation of proteins in the film matrix. FTIR spectra in the range of 1800–800 cm^−1^ were collected using a Spectrum 100 mid-IR instrument (Perkin Elmer, Shelton, CT, USA) equipped with an attenuated total reflection (ATR) accessory.

### 4.6. Equilibrium Water Swelling Study

The water uptake properties of the fabricated films were evaluated using an equilibrium water-swelling study. First, triplicate samples of the dehydrated (freeze-dried) gels were weighed and submerged in Milli-Q water. The water-submerged gel was periodically removed, excess water on the film surface was wiped off, and it was weighed and then submerged once more. This procedure was carried out repeatedly until the film’s weight became constant. The water uptake capacity (*h*) and crosslinking density (*v_e_*) of the fabricated film were calculated using the following equations [67]:

(2) $$h=\frac{W_{s\;\;}-W_{P\;}}{W_{P\;}}\times 100$$

(3) $$v_{1,s}=\frac{\frac{W_{P\;}}{\rho_{P}}}{\frac{W_{s\;\;}-W_{P\;}}{\rho_{2}}+\frac{W_{P\;}}{\rho_{P}}}$$

(4) $$v_{e}=\frac{-\ln\left(1-v_{1,s}\right)+v_{1,s}+Xv_{1,s}}{V_{2}[v_{1,s}-\frac{1}{2}v_{1,s}]}$$

where *W_s_* is the weight of the equilibrium water swollen film and *W_p_* is the weight of dehydrated or dry gel. The volume fraction (*v*_1,*s*_) represented the equilibrium swollen state of the film. The density of the dehydrated gluten gel was measured to be 1.42 g/cm³ (*ρ_p_*), while a density of 0.997 g/cm³ (*ρ*_2_) was used for water. The interaction parameter between gluten and water in the Flory–Huggins model was represented as *X* and assumed to be 0.95. Furthermore, the molar volume of water (*V*_2_) was determined as 18 cm³/mol [9,10].

### 4.7. Scanning Electron Microscopy (SEM)

The cross-sectional morphology and pore structure of the freeze-dried gel were examined using the Quanta 200 SEM (FEI, Hillsboro, OR, USA). The samples were sputter-coated with 10 nm of platinum for analysis.

### 4.8. Small-Angle and Ultrasmall-Angle Neutron Scattering

The hierarchical structure of the equilibrium water-swollen protein film was analyzed using the Quokka small-angle neutron scattering (SANS) [68] and the Kookaburra ultrasmall-angle neutron scattering (USANS) [69] instruments at ANSTO. The neutron scattering length density (SLD) of the hydrogenated protein sample is approximately 2.05 × 10^−6^Å^−2^, which provides good neutron contrast against the isotope of water, and deuterium oxide (D_2_O) had an SLD of 6.36 × 10^−6^Å^−2^ [70]. Therefore, the fabricated films were dialyzed against D_2_O and loaded with D_2_O in Quokka demountable cells (20 mm diameter and 1 mm path length) with a quartz window for both SANS and USANS measurements. The neutron scattering data were collected in the scattering vector (q) range of 0.0007 to 0.7 Å for SANS (using a neutron wavelength of 8.1 Å–for lens optic configuration with a sample–detector distance of 20 m, and 5.0 Å for sample–detector distances of 12 and 1.3 m), and 0.00005 to 0.003 Å for USANS (using a neutron wavelength of 4.7 Å). The obtained SANS data were reduced (using Quokka macros), background subtracted, and combined as reported in our previous publications [33,34]. USANS data were also background subtracted and desmeared using gumtree and mantid software (version 5.1), respectively [71,72]. Desmeared USANS data were then merged with SANS data using Quokka macros on IGOR Pro software (version 8.0). The structural parameters of samples were determined by fitting desired model functions to SANS data using the SasView computer program.

### 4.9. Rheology

The rheological properties of equilibrium water-swollen films were measured using the Discovery HR-03 rheometer (TA Instruments, New Castle, DE, USA) equipped with an 8 mm parallel plate accessory. The viscoelastic properties were measured as a function of oscillation strain (1–100%) and angular frequency (1–100 rad/s) at a fixed temperature of 25 °C.

### 4.10. Differential Scanning Calorimetry (DSC)

The thermal properties of fabricated films and precursor protein powders were investigated using a Discovery DSC thermal analyzer (TA Instruments, New Castle, DE, USA). The samples were sealed in Tzero hermetic aluminum pans, and the measurement was performed under a nitrogen atmosphere (50 mL/min flow) at temperatures of 25 to 350 °C with a heating rate of 10 °C/min.

### 4.11. Hydrolytic Degradation Study

The hydrolytic degradation profile of the fabricated films was studied at 25 °C by immersing the film samples in distilled water and periodically measuring their weight changes over time.

### 4.12. 3D Printing

The printability of hybrid films using photocurable ink (comprising 14 wt.% gluten, 6 wt.% keratin, 5 mM Ru(BPY)_3_, and 28 mM APS) was investigated using a solution extrusion-based BioScaffolder 3D printer (GeSiM, Radeberg, Germany). The printing parameters were controlled using GeSIM Robotics software (version 1.15). The ink was loaded into a 10 mL syringe cartridge and extruded (with pressure in the range of 40–80 kPa) through a 23-gauge diameter nozzle. The printing process was performed with breaks of 0.1 s between the initiation and termination points and the strands were deposited onto a glass substrate using a layer-by-layer printing technique, with a printing speed ranging from 10 to 20 mm/s. Photocrosslinking of the printed ink was achieved by shining white light for 40 s per layer. The dimensional accuracy of the printed structures was evaluated (immediately after the printing and photocrosslinking) using Equation (5), where the printing accuracy is *P* [73,74]. The computed value was obtained by averaging four measurements taken from both the printed dimension (*D_i_*) and the experimental dimension (*D*), where the dimensions can be length, width, and thickness.

(5) $$P=\left(1-\frac{D_{i}-D}{D}\;\right)\times 100$$

## Figures and Tables

**Table 1:** Composition and water uptake results of fabricated films.

Sample Name	Gluten:Keratin (Weight Ratio)	Equilibrium Swelling Study		SEM
		Water Uptake Capacity (%)	Crosslink Density (×10^−3^ mol/cm^3^)	Pore Size (µm)
G20 gel	100:0	83.61 ± 3.76	0.365 ± 0.001	1.65 ± 0.41
G18K2 gel	90:10	105.59 ± 5.89	0.359 ± 0.001	3.42 ± 1.12
G16K4 gel	80:20	122.30 ± 5.49	0.355 ± 0.001	3.81 ± 1.13
G14K6 gel	70:30	181.64 ± 4.89	0.347 ± 0.000	4.63 ± 2.02

**Table 2:** Structural parameters of fabricated film obtained from SANS data fit.

Sample	SANS High-Q (Guinier-Porod 1 Fit)		SANS Mid-Q (Guinier-Porod 2 Fit)		SANS Mid-Q (Guinier-Porod 3 Fit)		SANS Low-Q (Power Law Fit)	USANS Very Low-Q (2π/Q)
	Porod Slope	Rg (Å)	Porod Slope	Rg (Å)	Porod Slope	Rg (Å)	Porod Slope	Pore Size (µm)
G20 gel	4.00 ± 0.01	10.00 ± 0.01	2.76 ± 0.07	20.99 ± 0.08	2.25 ± 0.04	59.03 ± 0.23	2.93 ± 0.05	~1.24
G18K2 gel	4.00 ± 0.01	10.00 ± 0.01	2.56 ± 0.05	20.40 ± 0.07	2.24 ± 0.04	58.80 ± 0.22	2.82 ± 0.03	~1.64
G16K4 gel	4.00 ± 0.01	10.00 ± 0.01	2.46 ± 0.04	20.37 ± 0.05	2.23 ± 0.05	58.69 ± 0.27	2.79 ± 0.02	~2.06
G14K6 gel	4.00 ± 0.01	10.00 ± 0.01	2.40 ± 0.02	20.35 ± 0.05	2.09 ± 0.04	58.59 ± 0.25	2.65 ± 0.03	~2.20

## Data Availability

The data presented in this study are openly available in the article.

## Supplementary Materials

The following supporting information can be downloaded at: https://www.mdpi.com/article/10.3390/gels10020136/s1, Figure S1: MALDI-TOF MS spectrum of keratin powder; Figure S2: The deconvolution of the amide-I spectral region of the FTIR spectra of fabricated hydrogels. Ref. [75] cited in the Supplementary Materials.
