# Supplementary material for: 3D-Printable Sustainable Bioplastics from Gluten and Keratin
Source: Gels. 2024 Feb 7;10(2):136. doi: 10.3390/gels10020136 (PMC10887891; doi:10.3390/gels10020136)
Supplement: Supplementary file 1 [file gels-10-00136-s001.zip › gels-2823827-supplementary.pdf]

RMIT Classification: Trusted

Supplementary Materials

3D-Printable Sustainable Bioplastics from Gluten and Keratin

Jumana Rashid Mohammed Haroub Alshehhi[1], Nisal Wanasingha[1], Rajkamal Balu[1], Jitendra P. Mata[2], Kalpit Shah[1], Naba K. Dutta[1,*] and Namita Roy Choudhury[1,*]

[1]*Chemical and Environmental Engineering, School of Engineering, STEM College, RMIT University, Melbourne, VIC 3000, Australia*

[2]*Australian Centre for Neutron Scattering (ACNS), Australian Nuclear Science and Technology Organisation (ANSTO), Lucas Heights, NSW 2232, Australia*

*Author Correspondence: naba.dutta@rmit.edu.au (N.K.D.); namita.choudhury@rmit.edu.au (N.R.C.)

RMIT Classification: Trusted

S1. Matrix-assisted laser desorption ionization time-of-flight (MALDI-TOF) mass spectrometry.

The molecular weight of keratin protein was measured by MALDI-TOF analysis using a rapifleX ® MALDI instrument (Bruker). For sample preparation, about 5.0 mg of keratin powder and 5.0 mg of a-cyano-4-hydroxycinnamic acid (matrix) were first separately dissolved in 1 ml of solution mixture made of acetonitrile and water (1:1 weight ratio) containing 0.1% trifluoroacetic acid (TFA) [1]. About 10 µL of both sample and matrix solutions were mixed well, and 1 µL of the mixture was dispensed onto a MALDI plate and dried. The mass spectrum of the sample (with 10000 shots collected) was measured in the range of 500 to 5000 m/z (as shown below).

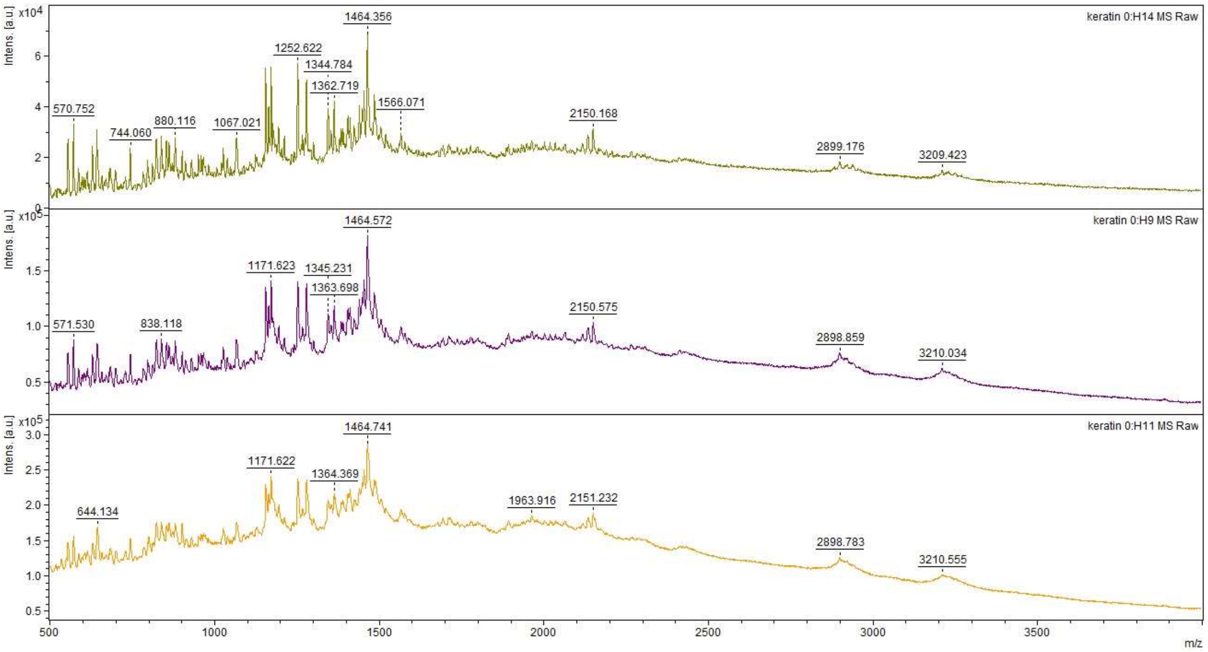

Figure S1: MALDI-TOF MS spectrum of keratin powder.

S2. Deconvolution of Fourier transform infrared (FTIR) spectroscopy data.

The secondary structure of proteins in fabricated hydrogels was estimated by deconvoluting the amide-I region (1580-1690 cm^{-1}) of the measured FTIR data (as shown below) using the MagicPlot Student software. The data was fit with multiple Gaussian peaks corresponding to β-sheets (1684 cm^{-1}), β-turns (1671 cm^{-1}), random coil and α-helices (1650 cm^{-1}), intramolecular β-sheets (1631 cm^{-1}), intermolecular β-sheets (1615 cm^{-1}), and glutamine side chain. (1596 cm^{-1}) [2]. The percentage of secondary structures were calculated from obtained peak areas.

RMIT Classification: Trusted

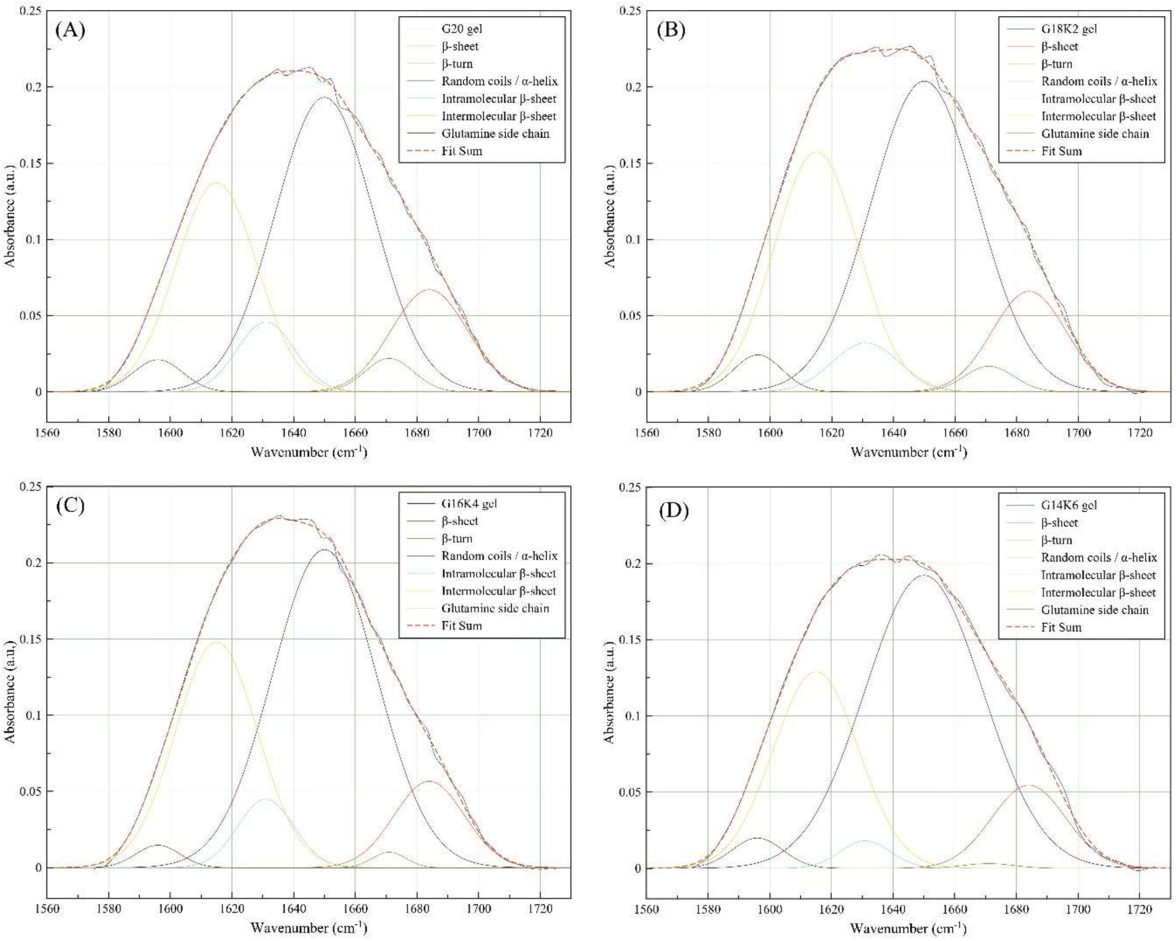

Figure S2: The deconvolution of the amide-I spectral region of the FTIR spectra of fabricated hydrogels.

Reference:

1. Cardamone, J. M. Investigating the microstructure of keratin extracted from wool: Peptide sequence (MALDI-TOF/TOF) and protein conformation (FTIR). *J. Mol. Struct.* **2010**, *969*, 97–105. https://doi.org/10.1016/j.molstruc.2010.01.048.
2. Georget, D. M. R.; Belton, P. S. Effects of temperature and water content on the secondary structure of wheat gluten studied by FTIR spectroscopy. *Biomacromolecules* **2006**, *7*, 469–475. https://doi.org/10.1021/bm050667j.
